# Peptide de novo sequencing of mixture tandem mass spectra

**DOI:** 10.1002/pmic.201500549

**Published:** 2016-08-05

**Authors:** Vladimir Gorshkov, Stéphanie Yuki Kolbeck Hotta, Thiago Verano‐Braga, Frank Kjeldsen

**Affiliations:** ^1^ Department of Biochemistry and Molecular BiologyUniversity of Southern Denmark Odense M, OdenseDenmark; ^2^ Department of Physiology and BiophysicsFederal University of Minas Gerais Belo Horizonte – MG, Belo HorizonteBrazil

**Keywords:** Bioinformatics, Complementary ions, De novo sequencing, Mass spectral interference, Mixture fragmentation spectra

## Abstract

The impact of mixture spectra deconvolution on the performance of four popular de novo sequencing programs was tested using artificially constructed mixture spectra as well as experimental proteomics data. Mixture fragmentation spectra are recognized as a limitation in proteomics because they decrease the identification performance using database search engines. De novo sequencing approaches are expected to be even more sensitive to the reduction in mass spectrum quality resulting from peptide precursor co‐isolation and thus prone to false identifications. The deconvolution approach matched complementary *b‐*, *y‐*ions to each precursor peptide mass, which allowed the creation of virtual spectra containing sequence specific fragment ions of each co‐isolated peptide. Deconvolution processing resulted in equally efficient identification rates but increased the absolute number of correctly sequenced peptides. The improvement was in the range of 20–35% additional peptide identifications for a HeLa lysate sample. Some correct sequences were identified only using unprocessed spectra; however, the number of these was lower than those where improvement was obtained by mass spectral deconvolution. Tight candidate peptide score distribution and high sensitivity to small changes in the mass spectrum introduced by the employed deconvolution method could explain some of the missing peptide identifications.

**Abbreviations**
LD: Levenshtein distance
MWCO: molecular weight cutoff
PSM: peptide‐spectrum match
SNP: single nucleotide polymorphism

## 1 Introduction

The most common method of analyzing proteomics data involves searching against protein sequence databases (Uniprot 1, NCBI GenBank 2, etc.) that accumulate protein and genetic information from numerous experimental sources 1. Bioinformatics programs were developed to correlate experimental data with database information include MASCOT 3, SEQUEST 4, OMSSA 5, X!Tandem 6, and MS‐GF+ 7. Although protein database searching has become reliable and routine in proteomics, it suffers from some shortcomings 8. First, protein sequences in the databases can be incomplete due to the absence of data from some biological species or chemical modifications of the proteins such as post‐translational modifications (PTMs) 9. Second, most eukaryotic proteins undergo alternative splicing of genes and may be subject to single nucleotide polymorphisms (SNPs) or amino acid mutations resulting in creation of proteoforms 10. Finally, only a portion of a known database sequence is used, e.g. the sequence is limited to certain biological species and protein modifications; therefore, some errors are possible if the sample contains proteins and peptides from other biological species and/or any unexpected PTMs 11.

**Significance of the study**

De novo sequencing methods are popular for reliable proteoform recognition and detection of point mutations in protein sequences that are crucial for truly personalized medicine. Wider application of de novo approaches requires high data quality. Improvements to the de novo sequencing software or data handling, therefore, should be of interest to the proteomics community. We hope that this study will raise awareness of the challenge of mixture fragmentation spectra introduced to data analysis. We demonstrated that better handling of mixture fragmentation spectra provided not only higher quality data but also increased the depth of the analysis.

De novo sequencing might be considered to be the most efficient method for protein sequencing because it allows deduction of peptide sequences directly from mass spectra not limited to database size. Most modern de novo sequencing programs convert the mass spectrum into a spectrum graph where the optimal path in this graph represents the peptide sequencing results 12, 13 obtained by dynamic programming 14, 15, 16, 17 or integer linear programming 18, 19. Probabilistic modeling 20, machine learning techniques 21, 22, composition restriction by accurate mass measurements 23, 24, divide‐and‐conquer 25, or linear sequencing algorithms 26, 27 are other methods used. Some well‐known programs are SHERENGA 12, Lutefisk 28, NovoHMM 20, PEAKS 16, PepNovo 29, and pNovo 30.

De novo sequencing approaches have recently received more attention due to the growing availability of high mass accuracy instrumentation 31. The best de novo algorithms are orders of magnitude faster than the fastest database search engines when used on large databases, however, they are less accurate 32. Thus, the most advanced modern sequencing software is reported to correctly determine peptide sequences in 30–50% of the mass spectra 13, 32, 33. The limited accuracy stems from incomplete series of sequence specific fragments often masked by peaks generated from various interferences 34. In addition, peptides with similar fragmentation spectra (“homeometric peptides”) 35 add to the sequencing complexity. Thus, selection of peptide relevant product ions and other methods of spectra processing used before de novo sequencing are important and several methods have been proposed specifically for this task 36, 37.

Mixture fragmentation spectra produced by simultaneous fragmentation of several co‐isolated peptide precursors are recognized by the proteomics community as a limitation in shotgun proteomics. Mixture spectra decrease the identification performance of database search engines 38, 39, 40, 41. De novo sequencing approaches are expected to be even more sensitive to the quality of the fragmentation spectrum and thus more prone to errors caused by peptide precursor co‐isolation. For the algorithms used for protein sequence database searching, the risk of misidentification is not as dramatic, particularly for protein targets without PTMs, because the search space of actual protein sequences is smaller than the set of sequences that can be theoretically generated 42. We previously reported on a data processing approach capable of deconvolution of mixture spectra using the principle of *b‐, y‐*ion complementarity 40, 41. Here, we investigated the impact of mixture spectra on the performance of commonly used de novo sequencing programs and evaluated the applicability of our approach to assist de novo sequencing. Given the diversity of de novo sequencing programs, we focused our comparison on the data processing, rather than benchmarking the programs.

## 2 Materials and methods

Extended experimental details can be found in the Supporting information.

### 2.1 HeLa cell lysate preparation and analysis

The human cervix epithelial adenocarcinoma (HeLa) cell lysate was prepared as published earlier 40, excluding the dimethyl labeling step. The lysate was chromatographically separated using a Dionex (Thermo, USA) Ultimate 3000 nanoUPLC system, coupled to an Orbitrap Fusion (Thermo Scientific, CA, USA) mass spectrometer. Peptides were fragmented using CID in the linear ion trap and analyzed in Orbitrap. Data analysis was performed using Thermo Proteome Discoverer 2.0.0.802, MASCOT 2.3 as the database search engine, and Percolator 2.05 43 to validate peptide‐spectrum matches (PSMs).

### 2.2 *Tityus serrulatus* venom sample

Collection and preparation of *T. serrulatus* venom was described previously 44. The lysate was separated using a Proxeon EASY nanoHPLC system coupled to an Orbitrap Velos Pro mass spectrometer (Thermo Scientific, CA, USA). Ions were isolated and fragmented using CID in the linear ion trap followed by Orbitrap detection.

### 2.3 Artificial mixture spectra creation

All data manipulation was performed using developed scripts in Python (v. 2.7.6). High‐quality PSMs with unique sequences were selected from HeLa cell lysate analysis and used to form 5000 artificial mixture spectra each consisting of two merged peptide fragment spectra having the absolute difference between parent ions mass‐to‐charge values less than one unit. Mimicking data‐dependent acquisition experiment, one fragment spectrum of each peptide pair was considered target and another contaminating peptide (target peptide corresponds to the peptide targeted/selected by the mass spectrometer for fragmentation, while contaminant peptide corresponds to the peptide sharing mass and time space with the target one, though not selected by the mass spectrometer as the progenitor of the fragmentation spectrum). Corresponding spectra were cleaned of any interfering peaks and mixed preserving total ion intensity with the following mixture ratios between targets and contaminating peptides: 0.001, 0.01, 0.05, 0.1, 0.25, 0.5, 0.75, 0.9, 0.95, 0.99, and 0.999. Spectra were saved in MASCOT generic format for further processing. In addition, the data set having 50% mixture and 50% non‐mixture spectra was created. Additional details could be found in Extended Materials and Methods and Fig. S1 of the Supporting Information.

### 2.4 Mixture spectra deconvolution

Mixture spectra deconvolution was performed as reported earlier 40. Proteome Discoverer was used for mass spectra processing. First, spectra were charge deconvoluted (all ions singly charged) using MSn Deconvolution node followed by mixture spectra deconvolution using Complementary Finder node. For complete HeLa lysate and scorpion samples, considered masses for co‐isolated peptides were required to have peptide mass peaks in the corresponding parent mass spectrum, and no restrictions (e.g., intensity, member of an isotopic cluster, etc.) were applied to the mass peak. All masses for co‐isolated peptides were accepted during the analysis of artificial mixture spectra. Typical processing time on a regular benchtop computer was less than a minute for artificial mixture spectra and scorpion samples and under 10 min for complete HeLa lysate. Mixture spectra deconvolution resulted in the generation of two additional spectra files for each input file. The first (purified spectra) contained only target spectra excluded from contaminating fragments, extracted co‐isolated spectra were not added to the resulting file. The second (deconvoluted spectra) contained concatenated purified and extracted spectra.

### 2.5 De novo sequencing

The following programs were used for de novo sequencing: PEAKS (v7.0 build 20140912) 16, pNovo+ (v1.3) 30, pepNovo+ (v3.1 release 20101117) 29, 35, 45, 46 and Novor (v1.1) 47. The following parameters were used for all programs: parent mass tolerance – 10 ppm; fragment mass tolerance – 0.02 Da; enzyme – trypsin; carbamidomethylated cysteine and oxidized methionine as fixed and variable modifications, respectively. PEAKS specific parameters: max variable PTMs per peptide – 3; number of report peptides – 5. pNovo+ specific parameters: activation type – CID; minimal precursor mass – 300 Da; maximal precursor mass – 5000 Da. pepNovo+ specific parameters: precursor mass tolerance – 0.02 Da (pepNovo+ support only absolute tolerance for parent mass); no quality filter; model – CID_IT_TRYP; use spectrum precursor m/z and charge. The following score thresholds were used: PEAKS ALC > 66; pNovo+ Score > 30; Novor aaScore > 36 and no threshold for pepNovo+. Score thresholds were empirically determined and normalized to provide the closest possible accuracy of each de novo sequencing program. More details can be found in Extended Materials and Methods (Supporting information).

### 2.6 Database search of artificial mixture spectra

The following parameters were used by MASCOT (v. 2.3.2) and SequestHT (as included in Proteome Discoverer): parent mass tolerance – 10 ppm; fragment mass tolerance – 0.02 Da; enzyme – trypsin, full specificity; maximum missed cleavages – 2; carbamidomethylated cysteine and oxidized methionine as fixed and variable modifications, respectively.

## 3 Results and discussion

### 3.1 Comparison of mixture spectra impact on de novo and database sequencing

**Figure 1:**
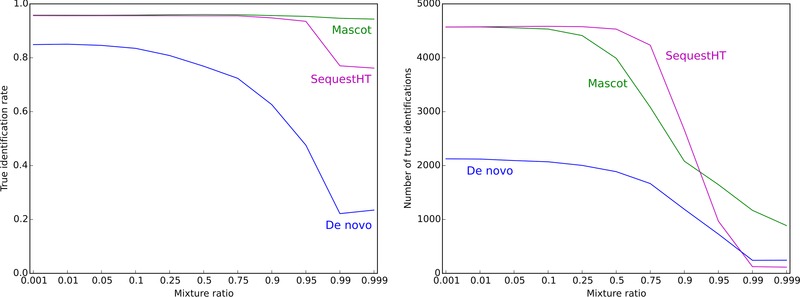
True identification rate and the number of true identifications at different mixture ratios for *de novo* (PEAKS) and database searching (MASCOT and SequestHT). Cutoff: IonsScore > 13 (MASCOT), XCorr > 1.0 (SequestHT), ALC > 66 (*de novo*); LD ≤ 2.

Artificially created mixture spectra served as an initial assessment of the effect of mixture spectra on de novo sequencing (see Extended Materials and Methods (Supporting Information) for preparation details). The formed datasets were analyzed by PEAKS de novo in comparison to database searching with MASCOT and SequestHT. By construction, we knew true sequences of the peptides forming each spectrum, which allowed for measurement of unbiased true identification rates. Those data are plotted in Fig. 1 for 11 tested mixture ratios. True identification rates were calculated as the fraction of all reported peptide sequences that matched either the target peptide sequence or the contamination peptide sequence. It is known that several common errors can occur during de novo sequencing, for example, isoelement substitutions (glutamine with glycine‐alanine pair and asparagine with glycine‐glycine) 48. Each of these errors produces sequences with Levenshtein distance (LD) 49 of not more than two from the original sequence, therefore, LD ≤ 2 mismatch between the reference and reported sequences was allowed.

Increasing mixture ratio lowered the performance of the de novo method drastically, although database searching was insensitive for mixture ratios up to 0.95. By contrast, Houel et al. 38 reported a reduction in true identification rate from 1.0 to 0.86 at 0.5 mixture ratio using database searching. Note that, first, mass spectra in this study were of very high quality and free of any interference which is not typical for proteomics datasets and, second, search parameters were limited using prior knowledge of the mass spectral construction. From Fig. 1, the de novo sequencing was more affected by the presence of mixture fragmentation spectra; moreover, it is critical to achieve as high true identification rate as possible, because erroneous sequences require more effort to eliminate in the context of de novo sequencing.

### 3.2 De novo sequencing of artificial mixture spectra: accuracy

**Figure 2:**
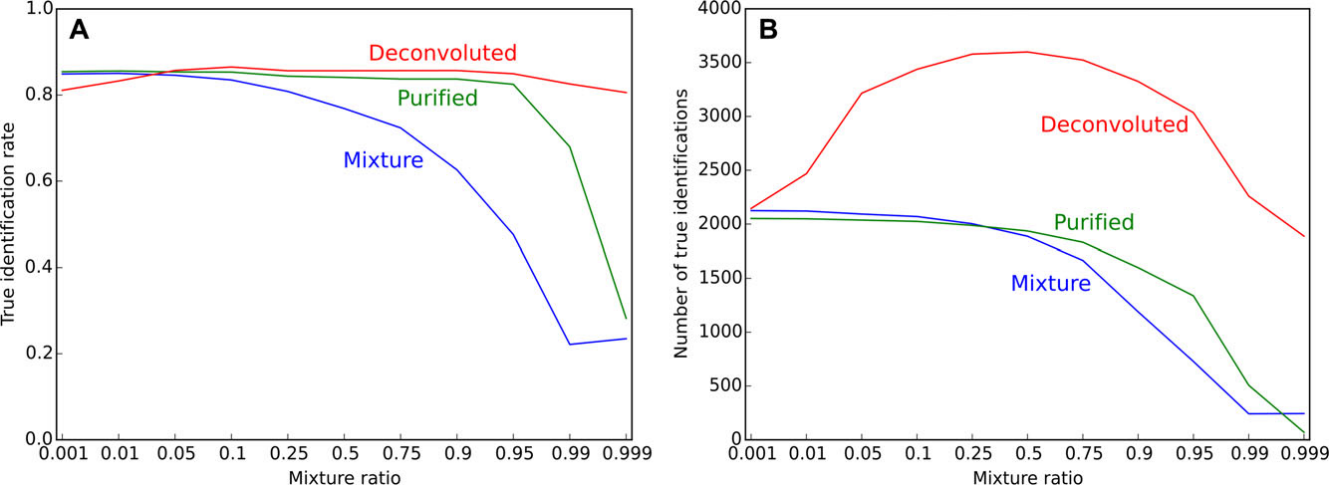
**(A)** True identification rate and **(B)** number of correct identifications for different processing of artificial mixture spectra. PEAKS ALC > 66; LD ≤ 2.

Mixture spectra were processed using MSn Deconvolution and Complementary Finder nodes 40. As a result, two additional spectra sets were created, the first (referenced as “purified”), contained spectra purified from any detected co‐isolated fragments, and the second (referenced as “deconvoluted”) contained concatenated purified spectra and extracted spectra of all detected co‐isolated peptides. Figure 2 illustrates the performance of PEAKS de novo depending on the mixture ratio. Elimination of contaminating fragments from target mass spectra (purified spectra) resulted in an enhanced true identification rate for mixture ratios below 0.95 (Fig. 2A). Performance degraded only for extreme mixture rates (>0.95), which might be due to the very low quality of the target component. For the deconvoluted datasets, the true identification rates were relatively constant for all mixture ratios. Again, some degradation in performance was observed for extreme ratios (<0.01 and >0.99) that could be explained by low ion abundance (low quality) of one of the extracted spectra, which increased the total number of reported peptide sequences, but could not provide enough mass spectral information for reliable sequence identification. Erroneously extracted spectra when no co‐isolation was present could also reduce true identification rates. Nevertheless, the total number of correct identifications (Fig. 2B) after deconvolution was higher than that found in the purified and mixture datasets.

The same analysis was performed for three other de novo sequencing programs (Supporting Information Fig. S3). To account for differences in scoring algorithms used by each program, we applied normalization of score cutoffs using a standard dataset (refer to Extended Materials and Methods and Fig. S2 in the Supporting Information for details). In spite of normalization, the observed performance still varied between programs indicating more fundamental differences among them. However, the overall trend in true identification rates was similar to that observed earlier: mixture spectra deconvolution produced constantly high accuracy independent of mixture rate. The number of true identifications followed the trend similar to PEAKS (Fig. 2B) for all programs, except for pNovo+. The latter delivered more identifications from unprocessed data compared to deconvoluted one for mixture rates lower than 0.25. This type of performance could probably be explained by the characteristics of this specific program; moreover pNovo+ delivered the smallest absolute number of identifications among all programs, thus score cutoff might be too restrictive.

### 3.3 De novo sequencing of artificial mixture spectra: number of identifications

**Figure 3:**
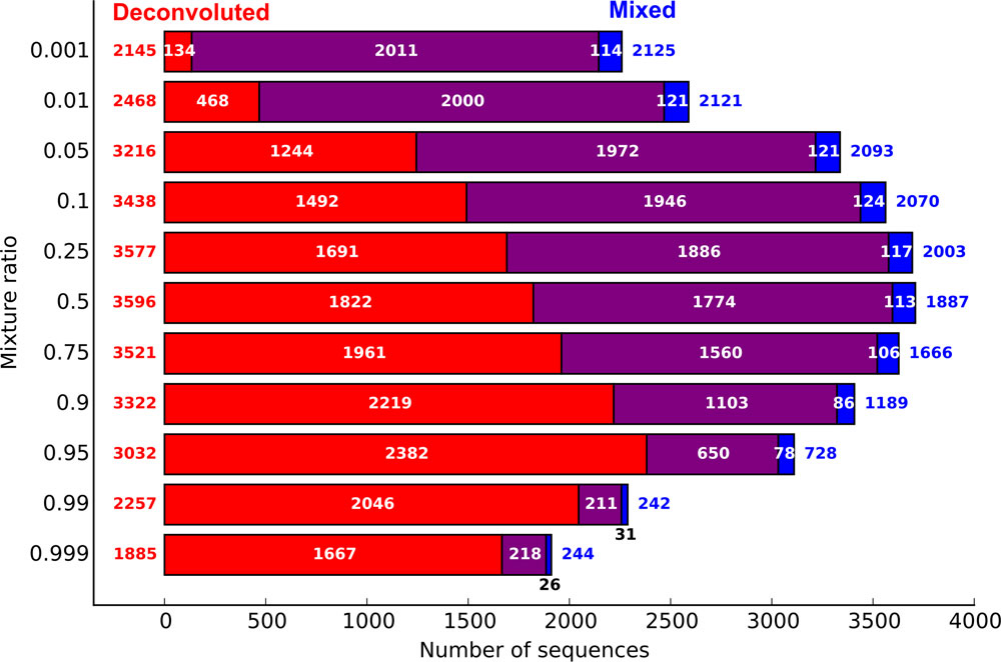
Number of correct sequences identified in artificial mixture spectra before and after mixture spectra deconvolution (PEAKS; ALC > 66). Red: sequences identified only in the deconvoluted dataset, blue: sequences identified only in the mixed dataset, purple: sequences identified in both datasets.

A comparison of correctly identified sequences at different mixture ratios is displayed in Fig. 3. Notably, the number of peptide sequences identified in the deconvoluted dataset increased with mixture ratio and reached the greatest absolute improvement at the mixture ratio of 0.95. The overlap between the results obtained for the mixture and deconvoluted datasets was high; however, at all mixture ratios, a small portion of the correct sequences was found only in the mixture dataset. This portion was as high as 5–6% at small and medium mixture rates (0.001 – 0.75) and reached 10–12% at mixture ratios > 0.95. It is expected that for some tandem mass spectra several possible sequences might obtain very similar scores, thus, some small differences in the spectrum (presence or absence of neutral loss peak) might favor one over others. These unintended changes could be introduced by the employed mixture spectrum deconvolution algorithm. However, for all mixture ratios the deconvoluted spectra resulted in a higher number of additionally identified sequences, which was achieved with better reliability (higher true identification rate).

The occurrence of mixture spectra in shotgun proteomics experiments is a function of both sample complexity and isolation capability of the mass spectrometer applied. However, recent reports 38, 39 showed that about 50% of mixture spectra are expected. We, therefore, created an artificial dataset containing 50% mixture spectra to simulate these conditions in a de novo setting. In total, 1881 correct sequences of 2311 sequences above the ALC threshold were obtained from the unprocessed dataset. In comparison, we found 2577 correct sequences of 3048 sequences above the ALC threshold for the deconvoluted dataset. This translated to success rates of 81 and 85% for unprocessed and deconvoluted datasets, respectively. These results demonstrated that the technique of mixture spectra deconvolution did not impair correct peptide sequence identification. Hence, deconvolution led to a larger absolute number of true identifications due to the interrogation of 37% more spectra. Similar to earlier observations, about 5% (83) of the correct sequences belonged solely to the unprocessed spectra; however, nearly one order of magnitude higher number of correct sequences (778) identified only from deconvoluted spectra compensated for this loss in identified peptides. Most sequences (1798) were identified by both processing methods.

### 3.4 De novo sequencing of HeLa cell lysate: accuracy

Encouraged by the results obtained with the artificially generated mixture spectra, we tested our deconvolution technique on the entire dataset of experimentally obtained CID MS/MS spectra from a HeLa lysate. Unlike the artificial dataset, this dataset included spectra with various abundances, noise levels, and non‐canonical fragments that complicate the de novo sequencing. Because it is important in de novo sequencing to have high spectral quality, we applied an additional filter for the mixture deconvolution processing. Considered co‐isolated peptide masses were required to have a corresponding peak in the parent spectrum (see Materials and Methods). The original spectrum file contained 137 893 MS^2^ spectra, which were converted to 215 362 MS^2^ spectra after processing; thus, the number of extracted spectra was close to the expected number of co‐isolations in a typical shotgun experiment (50%) 39.

Each tandem mass spectrum file was analyzed separately by de novo sequencing programs and resulting sequences were compared between unprocessed, purified target, and deconvoluted spectra. Since the exact peptide identity of each fragmentation spectrum cannot be known, and, therefore, true identification rate cannot be measured directly, the human protein database concatenated with common contaminants was used to estimate it. However, not all de novo derived sequences absent in the database were falsely identified and, vice versa, even if an identified peptide exists in the database the fragmentation spectrum it was deduced from could have been produced by some other peptide. Details of database validation could be found in the Extended Materials and Methods (Supporting Information). Table 1 contains information on the valid identification rates, as a ratio between sequencing results producing valid (present in the database) sequences and the total number of reported sequences above the score cutoff. The largest valid identification rate was observed for the purified spectra, whereas smaller rates were found for the mixed and deconvoluted spectra. Valid identification rates for deconvoluted spectra were slightly higher than for unprocessed data for PEAKS and pNovo+ and at the same level for Novor. The only exception was pepNovo+, where deconvolution resulted in lower valid identification rate. The latter observation can be explained by the fact, that we did not apply any score threshold for pepNovo+ results, in order to normalize its performance to the other programs.

**Table 1:** Valid identification rate by different processing of HeLa lysate sample

Program	Valid identification rate		
	Unprocessed (%)	Purified (%)	Deconvoluted (%)
PEAKS	46.18	49.80	47.58
pNovo+	45.63	52.59	51.74
pepNovo+	56.01	58.60	44.70
Novor	45.66	47.94	45.42

Novor aaScore > 36; pNovo+ Score > 30; PEAKS ALC > 66; pepNovo+ no restriction

At large, this suggests that our data processing eliminated some spectral interferences and improved the data quality of purified target spectra. However, co‐isolated spectra are expected to have limited ion statistics and, as the consequence, lower mass spectral quality, therefore, the overall performance decreases for the deconvoluted spectra.

### 3.5 De novo sequencing of HeLa cell lysate: number of identifications

**Figure 4:**
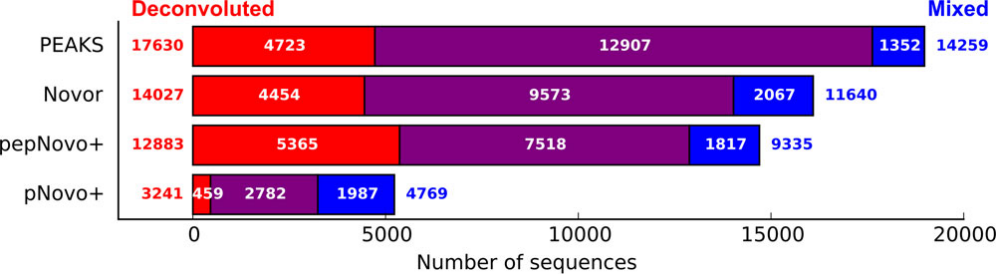
Number of valid sequences identified by different processing of HeLa lysate sample. Novor aaScore > 36; pNovo+ Score > 30; PEAKS ALC > 66; pepNovo+ no restriction. Red: sequences identified only in the deconvoluted dataset, blue: sequences identified only in the mixed dataset, purple: sequences identified in both datasets.

Only valid sequences were used to compare different processing methods. Both unprocessed and deconvoluted datasets resulted in largely the same peptide identifications (overlap > 80%), although the number of additional peptide identifications increased after deconvolution (Fig. 4). Similar to earlier observations, some valid sequences could only be found in the unprocessed dataset (ranging from 10 to 20% depending on de novo program employed). The observed increase in the number of sequences identified solely in the unprocessed dataset was expected due to lower data quality of many extracted peptide spectra. Again, pNovo+ was an exception with fewer identified sequences from deconvoluted data, although with a clear improvement in accuracy.

The difference in valid identification rate and number of peptide identifications was observed between the tested de novo sequencing programs (Table 1 and Fig. 4), which is in alignment with the results found for the artificial mixture dataset. However, for all programs, except pNovo+, improvement in the number of correct sequences with little to no degradation in sequencing accuracy was observed by applying mixture spectra deconvolution.

### 3.6 De novo sequencing of *T. serrulatus*

**Figure 5:**
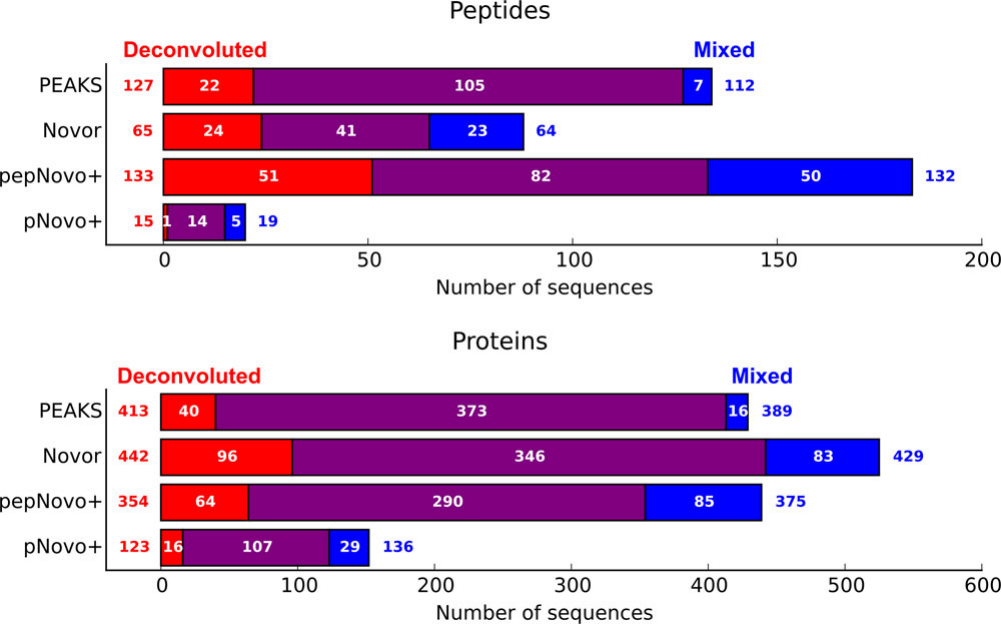
Number of sequences identified in scorpion samples. Novor aaScore > 36; pNovo+ Score > 30; PEAKS ALC > 66; pepNovo+ no restriction. Red: sequences identified only in deconvoluted dataset, blue: sequences identified only in mixed dataset, purple: sequences identified in both datasets. All identified sequences reported without validation.

Re‐analysis of the venom of scorpion *T. serrulatus* served as a test of our deconvolution approach with a sample of unknown protein/peptide composition. Two samples were prepared. One sample consisted of a non‐digested low molecular mass fraction (10 000 MWCO) and another sample consisted of a high molecular mass fraction of venom digested with trypsin: these samples are referenced as peptides and proteins, respectively. Because lower molecular complexity of the scorpion samples compared to the HeLa lysate sample was anticipated, we decided to analyze these samples by HPLC‐MS/MS‐CID with a short analytical gradient of 10 min in an attempt to retain realistic co‐isolation rate. Fig. 5 displays the number of identified sequences from unprocessed and deconvoluted datasets.

Deconvolution of mixture spectra typically resulted in some increase in the number of identified peptide sequences. As previously observed, the magnitude of the effect was different between de novo sequencing programs: for PEAKS the increase in peptide identification reached 13%, and little to no improvement was observed for other programs. The primary explanation for this might be found in the diversity in the sequencing algorithms and scoring schemes used by each program. For example, pepNovo+ uses fragmentation model trained on a large number of real spectra, however, the spectra produced after the deconvolution might not fulfill the assumptions of the model well. However, the results observed in the analysis of the HeLa sample were different; therefore, the explanation might also include the nature of the sample and employed instrumentation. In particular, the sample complexity was different between the two biological samples. Obviously, the effect of mixture spectra deconvolution was limited for less complex samples. This is in line with a lower number of extracted spectra for each input spectrum (0.12 and 0.28 for peptide and protein samples, respectively). The same number was 0.56 for the HeLa sample. Moreover, lower mass spectral quality and/or higher de novo sequencing complexity of non‐tryptic peptides was expected. Because tryptic peptides have a basic residue (lysine or arginine) at their C‐termini, they tend to produce more informative fragments than the non‐tryptic peptides. The overlap between unprocessed and deconvoluted spectra was similar to the analysis of HeLa lysate for the protein sample and lower than expected for the peptide sample. The opposite observation was made for the number of sequences identified only in the unprocessed sample. Although there were no solid criteria to validate resulting sequences, it was expected that the true identification rate was lower for non‐tryptic peptides, which explained both observations.

Verano‐Braga et al. 44 reported 317 peptides from 1225 different sequence tags, matching known genes of *Tityus sp*. detected by PEAKS (ALC > 70) in the peptide fraction of the venom. Our analysis detected 116 sequences of which 41 could be assigned to proteins of *Tityus sp*. (103 sequences matched 40 proteins without deconvolution) at the same score threshold. Observed numbers were less, however, because we used only CID fragmentation rather than a combination of CID, ETD, and HCD used in the above‐mentioned study 44. The repertoire of functional classes observed herein was close to that observed by Verano‐Braga et al. and included Hypotensins and Hypotensin‐like peptide, Pape peptide, and several potassium channel toxins. The only protein not present in the earlier report 44 was Venom Peptide 7 (P86828), which was identified with the single high scoring peptide (RLRSKG) in unprocessed and deconvoluted datasets (ALC 93 and ALC 96, respectively).

## 4 Concluding remarks

High data quality is one of the crucial requirements for successful de novo sequencing. The presence of peptide co‐isolation adds to the spectral complexity and hence it is desirable to develop efficient methods for mixture spectra deconvolution. Although programs to analyze mixture spectra are available 50, 51, 52, 53, they have not yet been evaluated in the context of de novo sequencing. Using artificial mixture spectra with various mixture ratios, we demonstrated that the true identification rate was affected by the presence of co‐isolated fragments in the mass spectrum, particularly for high mixture ratios. The observed degradation was more prominent, compared to database search methods, due to the larger search space tested by de novo approaches. Mixture spectra deconvolution was tested in combination with four de novo sequencing programs on three different datasets. The proposed deconvolution technique resulted in the identification of more peptide sequences without compromising the identification rates. The employed deconvolution approach was not without limitations and further improvement could make this technique even more efficient. In particular, a smaller fraction of correct peptide sequences was only identified in unprocessed datasets. Some of these missing identifications could be explained by the tight score distribution of candidate sequences resulting in high sensitivity to small changes in the mass spectrum introduced by the employed deconvolution method. For the venom samples of *T. serrulatus*, only a moderate increase in the number of detected sequences was observed. Insufficient sample complexity resulting in a low number of co‐isolations and less informative fragmentation of non‐tryptic peptides might be the primary reasons for the observed effect, the latter of which could be improved by advancing de novo sequencing programs.

With this study we hope to raise awareness of the challenge that mixture fragmentation spectra introduces to data analysis. Wider application of de novo approaches dictated by the growing interest in the study of protein variants, non‐sequenced proteins, the presence of unusual protein modifications, and deeper proteome interrogation requires higher data quality. More efficient data handling of mixture fragmentation spectra provides not only higher quality data but increases the depth of analysis. The employed mixture spectra deconvolution technique uses a basic principle of fragment complementarity to detect and separate co‐isolated peptides. We believe that its performance can be further improved by incorporation of other principles, for example, retention time alignment or advanced noise suppression.

*The authors have declared no conflict of interest*.

## Supporting information

As a service to our authors and readers, this journal provides supporting information supplied by the authors. Such materials are peer reviewed and may be re‐organized for online delivery, but are not copy‐edited or typeset. Technical support issues arising from supporting information (other than missing files) should be addressed to the authors.

Suppl. Figure S1

Suppl. Figure S2

Suppl. Figure S3

Supporting Information
