# Supplementary figures and images for: Peptide de novo sequencing of mixture tandem mass spectra
Source: Proteomics. 2016 Aug 5;16(18):2470–9. doi: 10.1002/pmic.201500549 (PMC5297990; doi:10.1002/pmic.201500549)

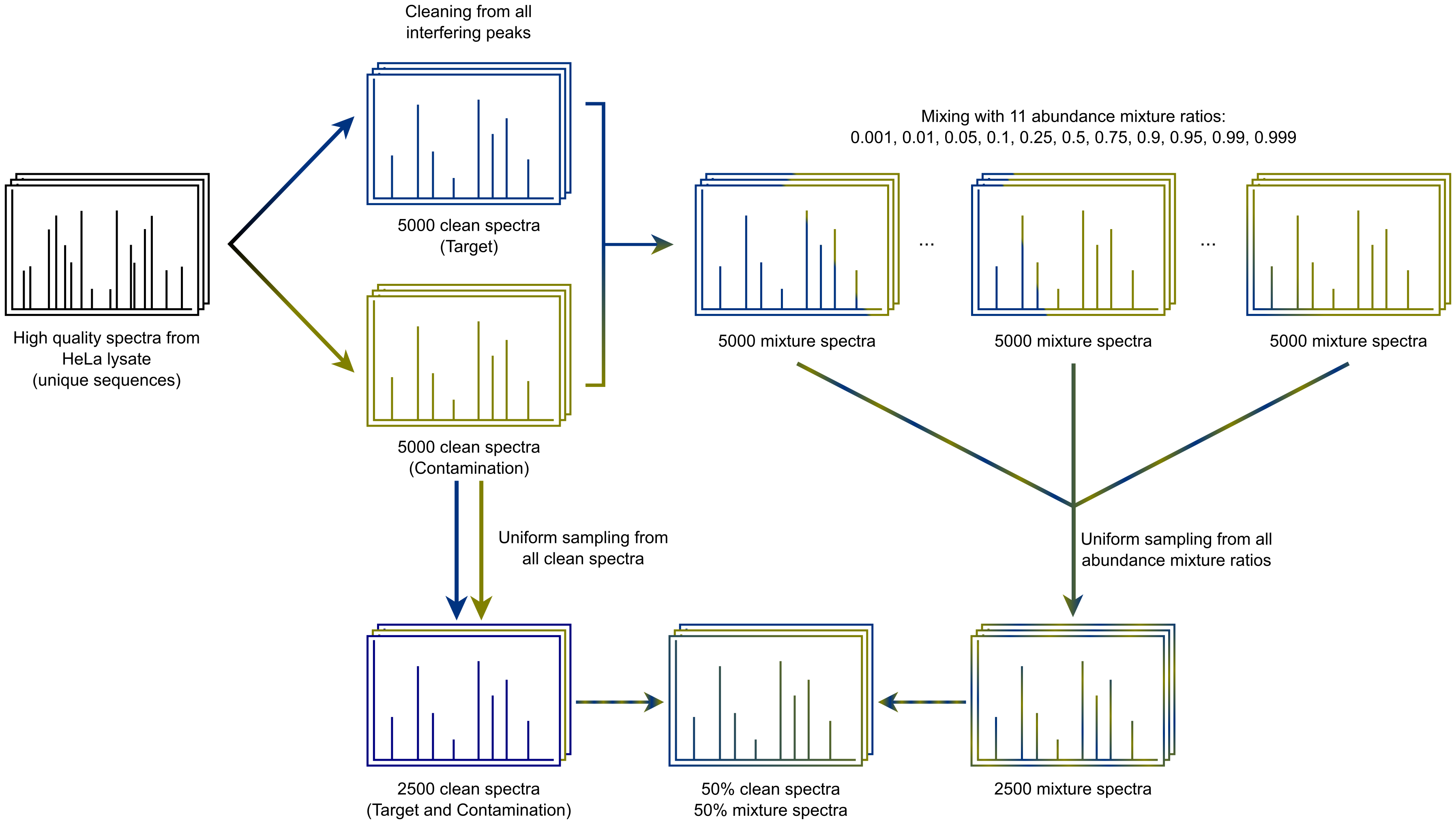

Supplement: Supplementary file 1 — Suppl. Figure S1 [file PMIC-16-2470-s001.tiff]

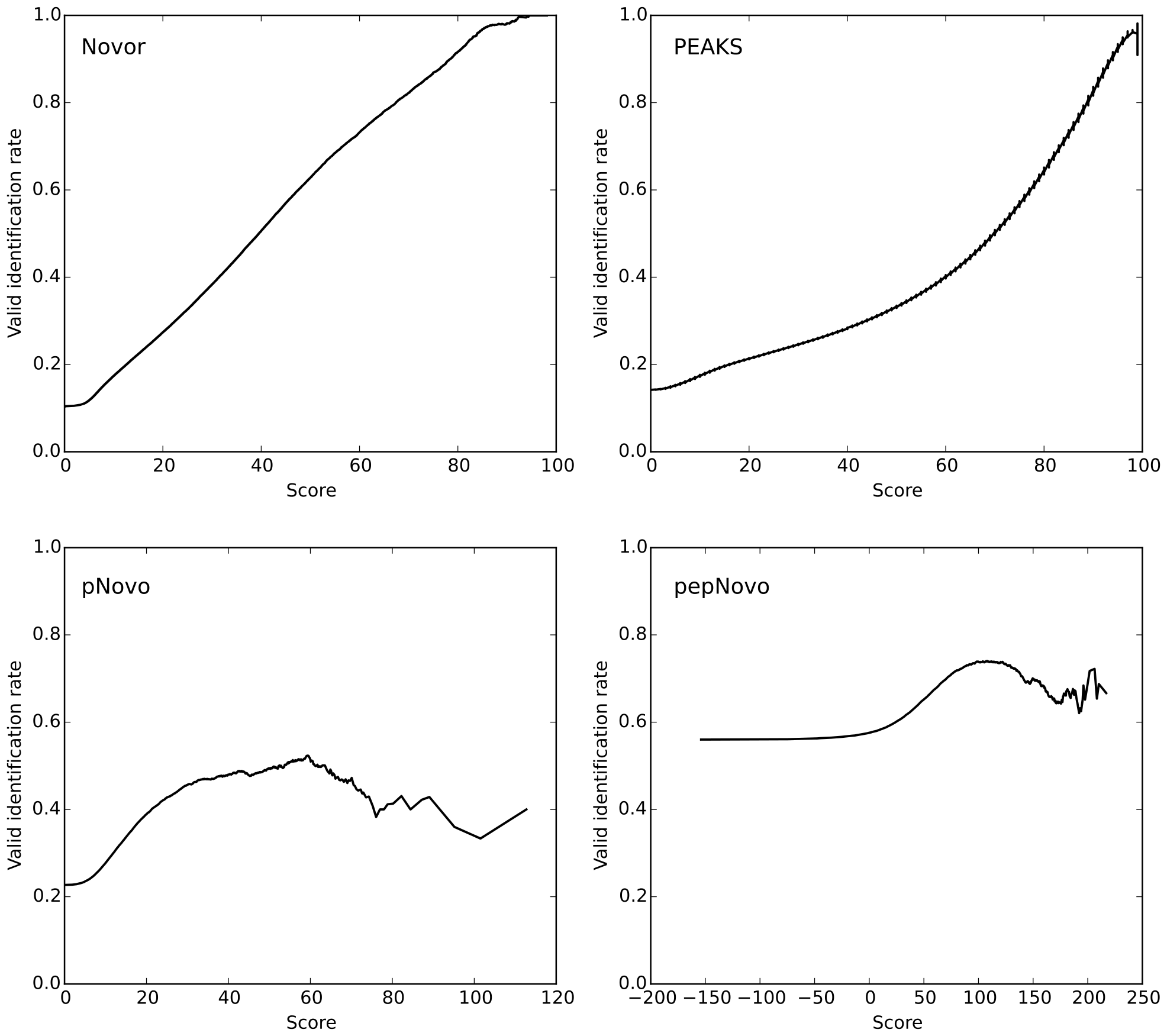

Supplement: Supplementary file 2 — Suppl. Figure S2 [file PMIC-16-2470-s002.tiff]

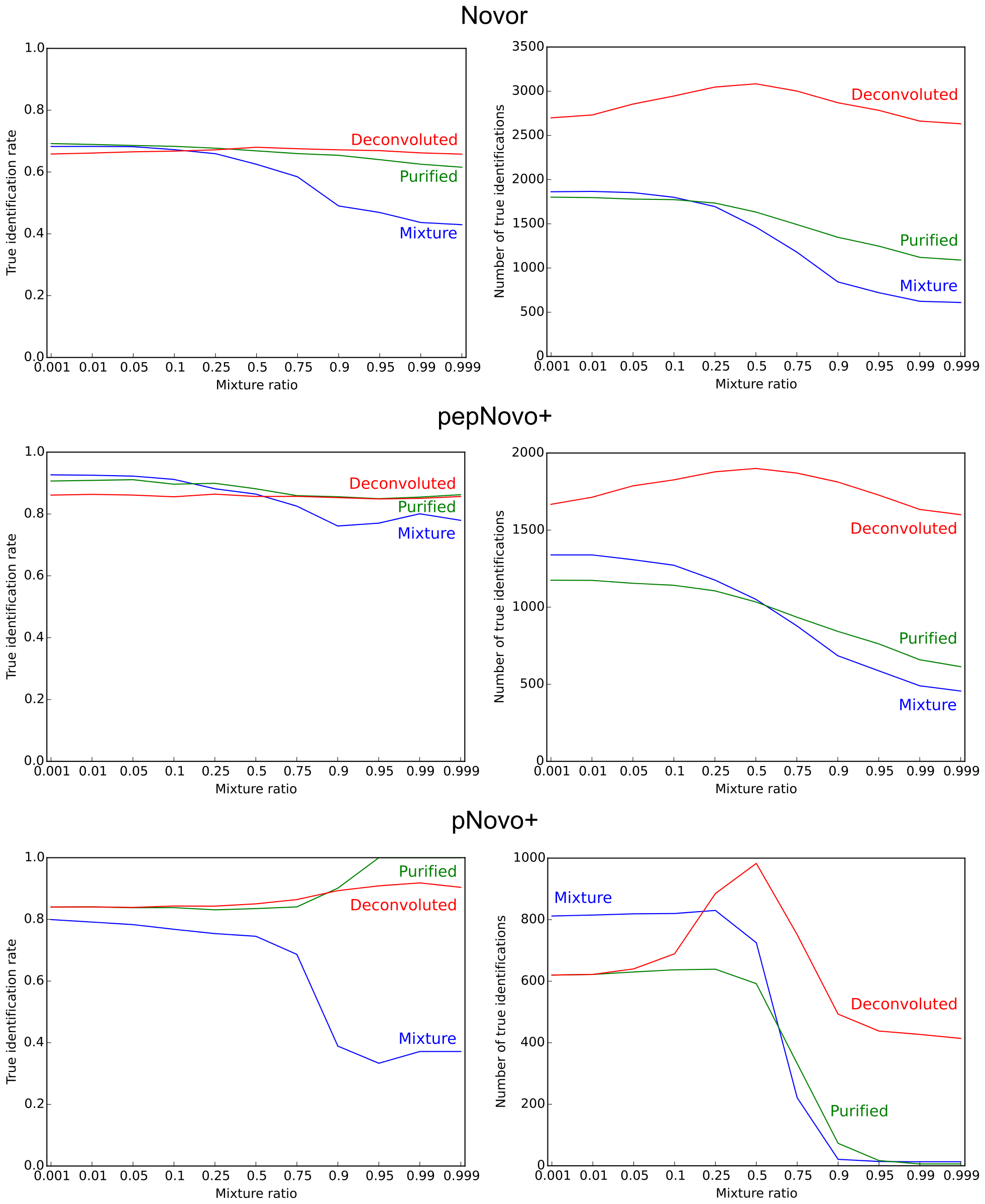

Supplement: Supplementary file 3 — Suppl. Figure S3 [file PMIC-16-2470-s003.tiff]
