# Supplementary material for: Peptide de novo sequencing of mixture tandem mass spectra
Source: Proteomics. 2016 Aug 5;16(18):2470–9. doi: 10.1002/pmic.201500549 (PMC5297990; doi:10.1002/pmic.201500549)
Supplement: Supplementary file 4 — Supporting Information [file PMIC-16-2470-s004.docx]

**Peptide *de novo* sequencing of mixture tandem mass spectra**

Vladimir Gorshkov^1*^, Stéphanie Yuki Kolbeck Hotta^1^, Thiago Verano-Braga^1,2^, Frank Kjeldsen^1^

^1^ Department of Biochemistry and Molecular Biology, University of Southern Denmark
Odense M, 5230, Denmark

^2^ Department of Physiology and Biophysics, Federal University of Minas Gerais
Belo Horizonte – MG, 31270-901, Brazil

^*^ Correspondence to Vladimir Gorshkov (vgor@bmb.sdu.dk, tel. +45 6550 8920)

**Table of contents**

**Extended Materials and Methods**

***Hela cell lysate preparation and analysis***

**Table S1.** **Hplc gradient used for the analysis**

***Tityus serrulatus venom sample***

***Artificial mixture spectra creation***

***Mixture spectra deconvolution***

**Figure S1.** Formation of artificial mixture spectra

***Database validation of HeLa de novo sequencing results***

***Scoring thresholds for different de novo programs***

**Figure S2.** Valid identification rate at different score thresholds

**Supplementary Figures**

**Figure S3.** True identification rate and number of correct peptides for different processing of artificial mixture spectra

**References**

**Extended Materials and Methods**

***HeLa cell lysate preparation and analysis***

Human cervix epithelial adenocarcinoma (HeLa) cells were cultured in 15 cm cell culture dishes in Dulbecco’s Modified Eagle Medium (DMEM) with Glutamax media supplemented with 10% FBS and 1% penicillin/streptomycin. Cells were harvested at 90%–95% confluency by scraping them off the plate followed by centrifugation. Pellets were stored at –80°C until further analysis.

Cells were lysed and proteins were on-filter digested as previously published [1]. Briefly, HeLa cells were lysed with a solution of 2% (w/v) SDS, 20 mmol/L TEAB, 0.1 mol/L DTT, phosphatase inhibitors (PhosSTOP, Roche, Switzerland), and protease inhibitors (cOmplete, Roche, Switzerland). Lysis was enhanced and DNA filaments sheared with tip sonication on ice. After appropriate dilution the protein concentration was measured using Qubit assay (Thermo Fisher Scientific, USA) as 4 μg/µL in the sample. Proteins were loaded by adding two times 75 µL (600 µg protein) on a spin-filter (Vivacon 500, 30,000 MWCO; Vivaproducts, USA). The SDS-buffer of the protein solution was washed using a urea-containing solution (8 mol/L urea, 20 mmol/L triethylammonium bicarbonate (TEAB)); 300 µL of the urea solution was used for washing after each loading followed by two washes with 200 µL urea and two washes with 375 µL of 1% (w/v) sodium deoxycholate (SDC), 20 mmol/L TEAB after both loadings. Alkylation of the reduced thiol groups was done with 50 mmol/L iodoacetamide, 1% (w/v) SDC, 20 mmol/L TEAB (300 µL of solution, followed by two times wash with 300 µL of 1% (w/v) SDC, 20 mmol/L TEAB), proteins were digested overnight with trypsin (1:100) (Promega, USA) in 1% (w/v) SDC, 20 mmol/L TEAB. Peptides were collected after centrifugation and SDC was removed using ethyl acetate and TFA (0.5% (v/v) final concentration).

Peptides were separated using a Dionex (Thermo, USA) Ultimate 3000 nanoUPLC system, coupled to a Thermo Orbitrap Fusion mass spectrometer. Peptides were focused on the precolumn (PepMap C18 10 cm x 150 µm i.d., 5 µm; Thermo, USA) and eluted from the analytical column (PepMap C18 50 cm x 75 µm i.d., 3 µm; Thermo, USA) with the gradient presented in Table S1. The mass spectrometer was operated in the 3 sec top-speed mode. MS^1^ spectra were recorded in the Orbitrap mass analyzer from 400 to 1200 Th, with 120,000 resolution at 200 Th, automated gain control (AGC) target value – 5e5, maximum accumulation time – 60 ms. Ions were isolated using a quadrupole mass filter with 2 Th wide isolation windows and fragmented using collision-induced dissociation (CID) in the linear ion trap. MS/MS spectra were acquired with Orbitrap detection with 15,000 resolution at 200 Th, AGC target – 1e4, maximum accumulation time – 40 ms.

**Table S1.** **Hplc gradient used for the analysis.**

A: 0.1% formic acid; B: 80% acetonitrile in 0.1% formic acid

| **Time (min)** | 0 | 5 | 25 | 205 | 245 | 270 | 285 | 287 | 300 |
| --- | --- | --- | --- | --- | --- | --- | --- | --- | --- |
| **B (%)** | 2 | 2 | 5 | 21 | 35 | 99 | 99 | 2 | 2 |

Data analysis was performed using Thermo Proteome Discoverer 2.0.0.802. Mascot 2.3 was used as the database search engine. SwissProt database (2014.04) restricted to *Homo sapiens* (20340 protein sequences) combined with a common contaminants database (231 protein sequences) was used. Search parameters were: parent ion mass tolerance – 5 ppm, fragment ion mass tolerance – 0.02 Th; fixed modifications – carbamidomethylated cysteine; variable modifications – oxidized methionine. Reversed decoy database was searched separately. Database search results were evaluated using Percolator 2.05 [2].

***Tityus serrulatus venom sample***

Collection and preparation of *Tityus serrulatus* venom was described previously [3]. Peptide samples were separated using a Proxeon EASY nanoHPLC system, coupled to a Thermo Orbitrap Velos Pro mass spectrometer (Thermo Scientific, CA, USA). Peptides were focused on a packed precolumn (5 cm x 100 µm i.d.) filled with Reprosil-Pur C18-AQ (5 µm, Dr. Maisch GmbH, Ammerbuch, Germany) and eluted from the analytical column (15 cm x 75 µm i.d.) filled with Reprosil-Pur C18-AQ (3 µm, Dr. Maisch GmbH, Ammerbuch, Germany) using a 10 min long (3 – 40% B) gradient. Mass spectrometer was configured to data-dependent acquisition, fragmenting the five most intense ion signals. MS1 and MS2 spectra were recorded in the Orbitrap mass analyzer using 60,000 and 15,000 resolution at 200 Th, respectively. Ions were isolated (2 Th window) and fragmented using collision-induced dissociation (CID) in the linear ion trap.

***Artificial mixture spectra creation***

All data manipulation was performed using developed scripts in Python (v. 2.7.6). We selected high quality peptide-spectrum matches (PSMs) having posterior-error probability (PEP) < 1∙10^–4^, as estimated by Percolator, and unique sequence (for all PSMs with the same sequence we selected only the one with the highest Mascot IonsScore) from HeLa cell lysate analysis. Next, 10,000 PSMs were selected randomly such as they formed pairs having the absolute difference between parent ion mass-to-charge values less than 1 unit. One member of each pair was considered target and another contaminating peptide (target peptide corresponds to the peptide targeted/selected by the mass spectrometer for fragmentation, while contaminant peptide corresponds to the peptide sharing mass and time space with the target one, though not selected by the mass spectrometer as the progenitor of the fragmentation spectrum). Only peaks that match corresponding *a*, *a–*H_2_O, *a–*NH_3_, *b*, *b–*H_2_O, *b–*NH_3_, *y*, *y–*H_2_O, *y–*NH_3_ ions in any probable charge state (from 1 to parent ion charge) with 10 ppm tolerance were preserved in the spectrum (up to 4 isotopic peaks for each of the ions were preserved). Total ion current (TIC), calculated as the sum of intensities of all ions in the spectrum, was normalized to 1∙10^5^, preserving all relations between ion intensities. We created 11 spectra sets modulating mixture rate between target and contamination component while preserving TIC, thus, each spectrum set had 5000 mixture spectra. The following mixture ratios were used: 0.001, 0.01, 0.05, 0.1, 0.25, 0.5, 0.75, 0.9, 0.95, 0.99, and 0.999. Spectra were saved in Mascot generic format (MGF) for further processing. In addition, a data set having 50% mixture spectra was created as follows: clean (uniform sampling from target and contamination spectra sets) and mixture spectra (uniform sampling from the 11 mixed spectra sets) sampled 1:1 (Figure S1).


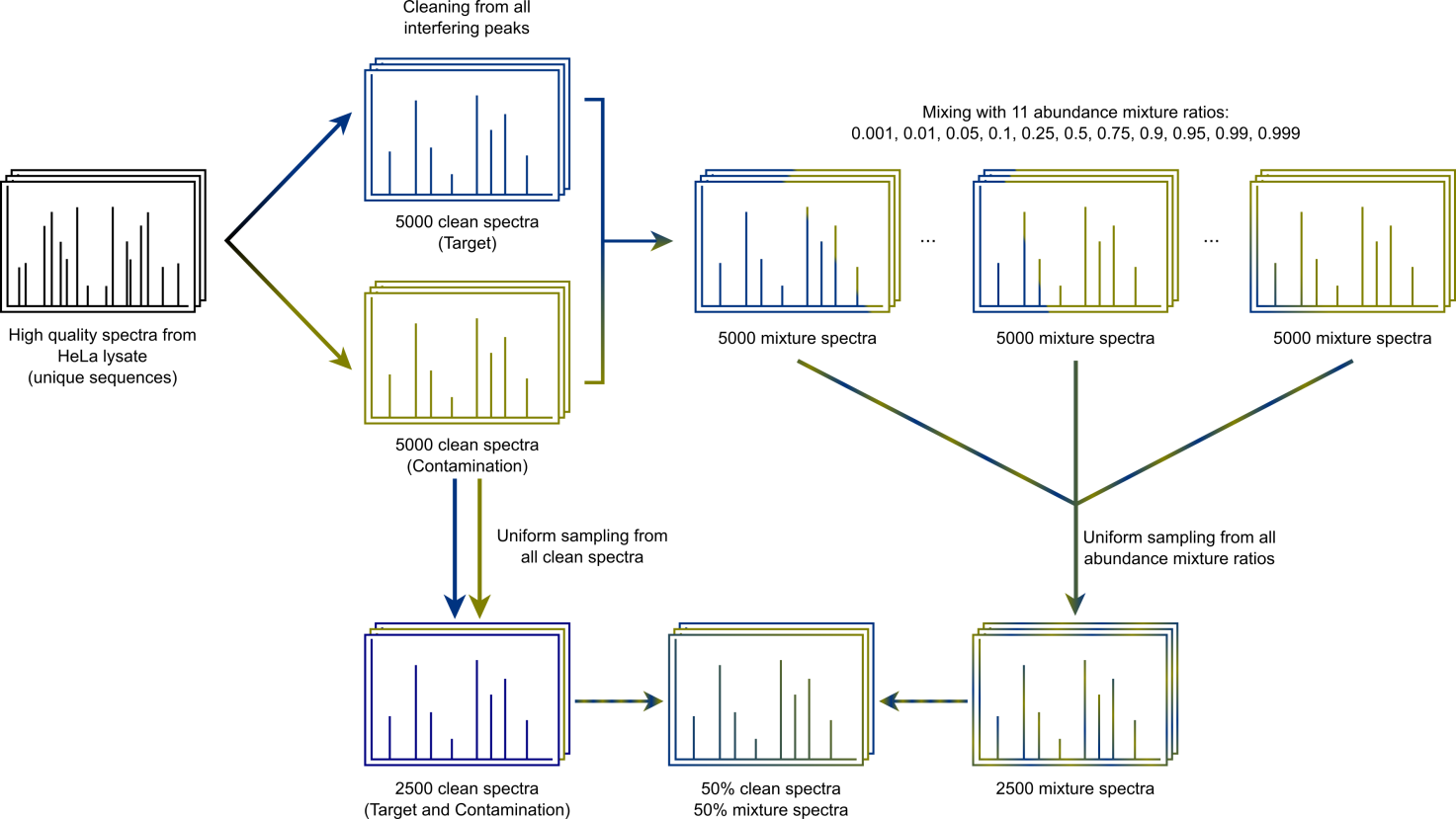


**Figure S1.** Formation of artificial mixture spectra.

***Mixture spectra deconvolution***

Mixture spectra deconvolution was performed as reported earlier [4]. Briefly, spectra files were processed in Thermo Proteome Discoverer 2.0.0.802 using MSn Deconvolution and Complementary Finder nodes. Spectra were charge deconvoluted (all ions singly charged), and all peaks that could not be assigned to any charge state according to the isotopic pattern were transferred to the deconvoluted spectra with charge state 1+. Complementary Finder node was applied with the following parameters: co-isolation window – 0.6 Th wider on the lower border than the isolation window; expected charge states for co-isolated ions 2+, 3+, and 4+; parent mass grouping tolerance – 5 ppm; minimal number of ions to extract co-isolated peptide – 6. For full HeLa lysate sample and scorpion samples allowed masses for co-isolated peptides were required to have peptide mass peaks in the corresponding parent mass spectrum (5 ppm tolerance), no restrictions (e.g. intensity, member of an isotopic cluster) were applied on the peptide mass peaks. All candidate peptide masses were accepted during the analysis of artificial mixture spectra. Two additional spectra files (MGF format) were generated for each input test file. The first one contained only target spectra excluded from contaminating fragments, while extracted spectra were not added to the resulting files, referenced later as purified spectra. The second one contained both purified and extracted spectra, referenced later as deconvoluted spectra.

***Database validation of HeLa de novo sequencing results***

Database of known *Homo sapiens* proteins (UniprotKB/SwissProt 2014.04, 20340 sequences) extended with common contaminants (231 sequences) was used to validate the *de novo* suggested peptide sequences. Substitution of isoleucine and leucine, since *de novo* sequencing cannot distinguish between these amino acids; substitution of asparagine with aspartate and glutamine with glutamate to account on deamidation and, finally, isoelement substitutions were allowed. When comparing sequences identified by different processing methods (unprocessed, purified and deconvoluted) LD ≤ 2 differences were considered the same sequence

***Scoring thresholds for different de novo programs***

Selecting the same score cutoff for each of the tested *de novo* sequencing programs was not trivial since they have essentially different scoring schemes. We balanced the score cutoffs with respect to the performance on the standard dataset (unprocessed HeLa lysate, validated against protein database as discussed earlier). Our aim was to find the score thresholds that delivered the most similar performance for each of the programs. The dependency between the score and the portion of valid sequences is represented in Figure S2. One can observe a significant difference between programs; Novor and PEAKS demonstrated the monotonous growth of accuracy with the score, while pNovo+ and pepNovo+ had the best accuracy for intermediate scores which decreased with higher scores. The highest valid identification rate for pNovo+ was 0.52 with the score cutoff of 59, however only about 0.009 of all reported peptides had a score higher than this value, therefore for our study we selected the score of 30 corresponding to slightly lower accuracy (0.46) but retaining 0.16 of all reported peptides. The scores for PEAKS and Novor were selected to match the performance of pNovo+, preserving 0.30 and 0.22 of all reported peptides, respectively. Since the lowest observed accuracy for pepNovo+ was 0.56, no score cutoff was applied to pepNovo+ results.


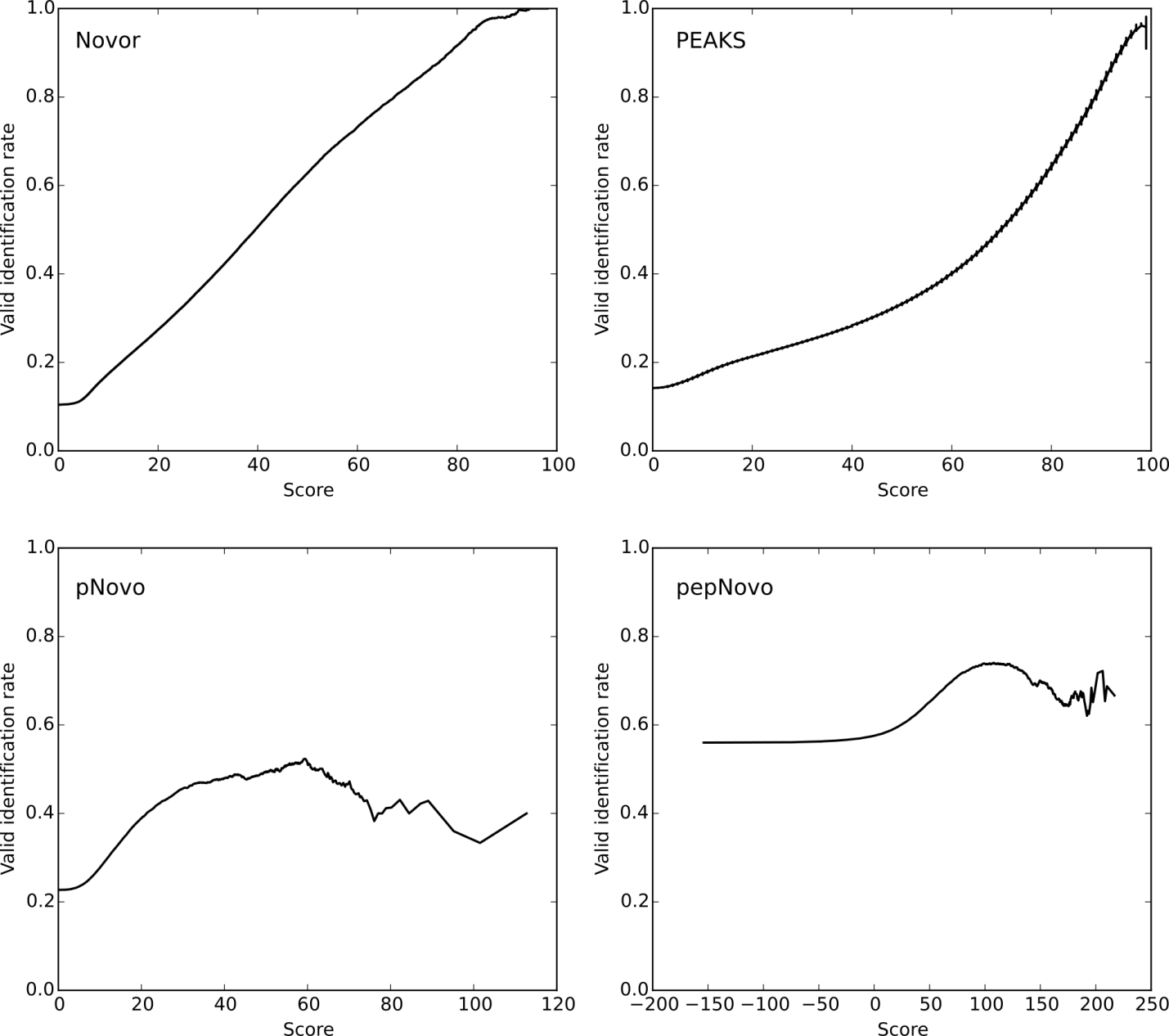


**Figure S2.** Valid identification rate at different score thresholds.

**Supplementary Figures**


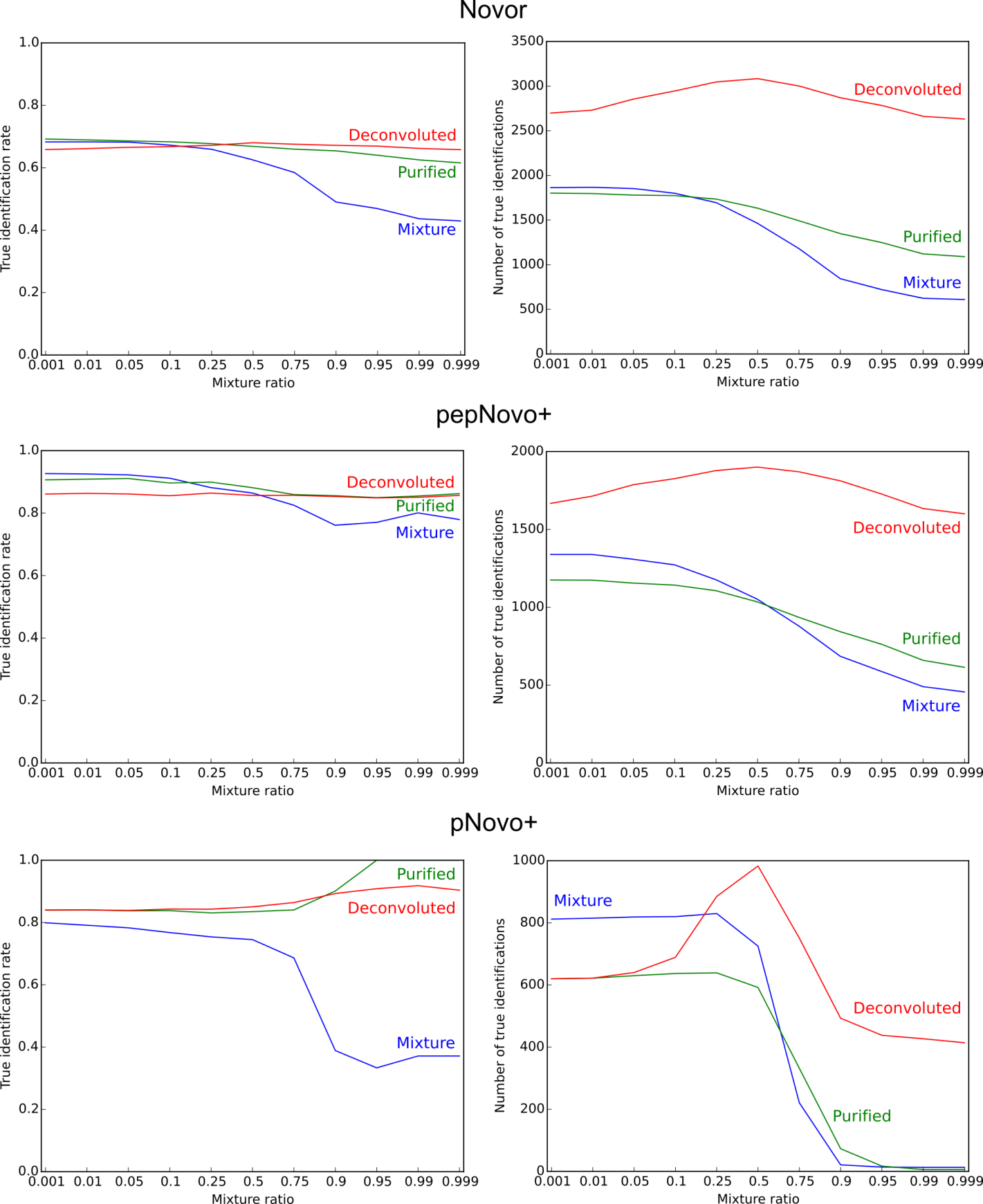


**Figure S3.** True identification rate (left column) and number of correct peptides (right column) using different *de novo* programs of processed and non-processed artificial mixture spectra.

Novor aaScore > 36; pNovo+ Score > 30; pepNovo+ no restriction; LD ≤ 2.

**References**

1. Leon, I.R., Schwammle, V., Jensen, O.N., Sprenger, R.R., Quantitative assessment of in-solution digestion efficiency identifies optimal protocols for unbiased protein analysis. *Mol Cell Proteomics* 2013, *12*, 2992-3005.

2. Kall, L., Canterbury, J.D., Weston, J., Noble, W.S., MacCoss, M.J., Semi-supervised learning for peptide identification from shotgun proteomics datasets. *Nat Methods* 2007, *4*, 923-925.

3. Verano-Braga, T., Dutra, A.A., Leon, I.R., Melo-Braga, M.N. et al., Moving pieces in a venomic puzzle: unveiling post-translationally modified toxins from Tityus serrulatus. *J Proteome Res* 2013, *12*, 3460-3470.

4. Gorshkov, V., Verano-Braga, T., Kjeldsen, F., SuperQuant: A Data Processing Approach to Increase Quantitative Proteome Coverage. *Anal Chem* 2015, *87*, 6319-6327.
